# Acupuncture Therapy for Cognitive Impairment: A Delphi Expert Consensus Survey

**DOI:** 10.3389/fnagi.2020.596081

**Published:** 2020-11-26

**Authors:** Xin-Tong Su, Li-Qiong Wang, Jin-Ling Li, Na Zhang, Lu Wang, Guang-Xia Shi, Jing-Wen Yang, Cun-Zhi Liu

**Affiliations:** ^1^International Acupuncture and Moxibustion Innovation Institute, School of Acupuncture-Moxibustion and Tuina, Beijing University of Chinese Medicine, Beijing, China; ^2^School of Acupuncture, Moxibustion and Tuina, Shandong University of Traditional Chinese Medicine, Jinan, China

**Keywords:** acupuncture, cognitive impairment, dementia, Alzheimer’s disease, vascular dementia, expert consensus

## Abstract

**Background:**

Current research evidence challenges clinical decision-making when acupuncture is taken into consideration in the treatment of cognitive impairment (CI). Aiming to provide some viable recommendations for acupuncture practitioners in dealing with actual clinic issues, an expert consensus study was conducted.

**Methods:**

A clinical question investigation among 47 acupuncturists yielded 24 initial items. Subsequently, systematic reviews on acupuncture for CI were searched within three online databases. A panel of 30 authoritative experts were requested to respond with agreement, neutrality, or disagreement for each item. Consensus establishment was defined as the percentage of agreement on a given item >80%.

**Results:**

Following a 2-round Delphi survey, there were 21 items reaching consensus and three items resulting in no consensus; of which 10 items reached 90∼100% agreement, and 80∼90% expert agreement was achieved for 11 items. These items could be roughly categorized into six domains: (1) therapeutic effects of acupuncture, (2) therapeutic principles, (3) acupoint selection and combination, (4) acupuncture parameters, (5) considerable combined therapies, and (6) possible adverse events.

**Conclusion:**

Without ready-made guidelines, this expert consensus may be conducive to guide acupuncturists in implementing clinical acupuncture practice for CI. Moreover, given the lack of high-quality research evidence and plenty of unresolved clinical issues in this field, it is of necessity to carry out more studies to better clarify the treatment algorithm.

## Introduction

Cognitive impairment (CI) is a common neurological disorder worldwide (Ferri et al., 2005). Nowadays, due to the global aging of the population, this disease brings health systems astronomical medical and socioeconomic burdens (Iadecola, 2013). In accordance with the report of the World Health Organization, the morbidity of CI will double every two decades and come up to more than 100 million by 2050 (Livingston et al., 2017). Alzheimer’s disease (AD) and vascular cognitive impairment (VCI) are the two main causes of CI (Hugo and Ganguli, 2014). Despite the emerging pharmacological treatments that have been developed, such as donepezil, memantine, and rivastigmine, which are widely recommended by the current guidelines (Sorbi et al., 2012; National Collaborating Centre for Mental Health, 2018), their therapeutic effects vary with different individuals and need further validation for certain types of CI (Van der Flier et al., 2018). Moreover, drug therapies always require long-duration use, which may result in adverse events and reduce the compliance of patients (Kavirajan and Schneider, 2007). Meanwhile, for the “predementia stage” such as subjective cognitive decline (SCD) and mild cognitive impairment (MCI), there is no drug effective for postponing the progression to dementia approved by the Food and Drug Administration (FDA) (Alzheimer’s Association, 2018). Therefore, more treatment options for CI urgently need to be explored.

As a main approach of Traditional Chinese Medicine (TCM) used in combating diseases for more than three millennia, acupuncture is a ubiquitously applied non-drug therapy with purported claims of effectiveness for CI in numerous Chinese hospitals (Peng et al., 2007; Wang et al., 2020). However, there is no established guideline for acupuncture in treating CI up to now. The concrete acupuncture protocols composed of acupuncture types and specific parameters, as well as acupoint selection and combination vary enormously in different literatures, which means that plenty of pragmatic issues, that acupuncture practitioners are concerned about, have not been answered. Owing to the deficiency in high-quality research evidence for drawing the confirmed conclusions, these complicated clinical issues may be arguably resolved to some degree by conducting an expert consensus survey (Hohmann et al., 2018a).

A consensus study can be performed scientifically with the assistance of the Delphi technique, which is a widely adopted structured process including the three main features: not requiring face-to-face contact, controlled feedback, and statistical expert response (Hasson et al., 2000; Hsu and Sandford, 2007; Hohmann et al., 2018b). As an iterative process, it can collect experts’ opinions from distinct stakeholders by a battery of surveys until either a consensus or an impasse has been reached (Hasson et al., 2000). As such, this method has been utilized successfully in the expert consensus surveys of acupuncture therapy previously (Trevelyan et al., 2015; Sun et al., 2020).

To provide viable recommendations on specific clinical issues and set up a standardized and optimized algorithm in treating CI for acupuncture practitioners, we convened a panel of Chinese authoritative acupuncture experts to perform a multi-round consensus survey with the help of the Delphi technique.

## Materials and Methods

**FIGURE 1:**
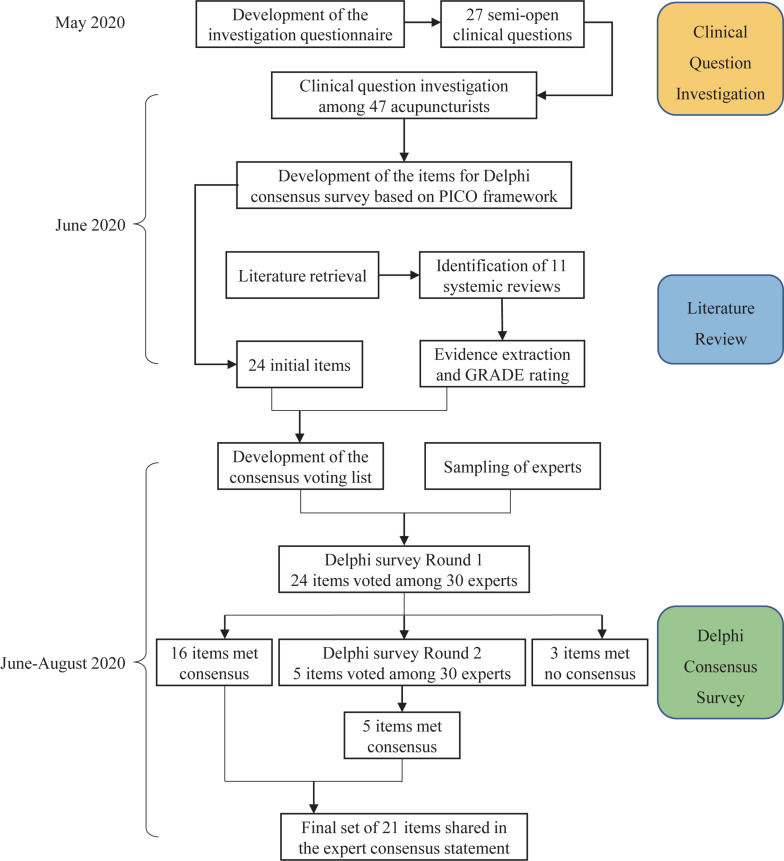
Work-flow diagram of the consensus development process.

A steering group was built in advance for drawing up the detailed schedule, providing operational guidance, and facilitating the entire process. The steering group was organized by one senior acupuncture expert (Cun-Zhi Liu), two clinical acupuncturists (Xin-Tong Su and Guang-Xia Shi), one methodological researcher (Li-Qiong Wang) and two research secretaries (Jin-Ling Li and Na Zhang). A summarized work-flow diagram of the study is shown in Figure 1.

The study was conducted and reported in reference to the corresponding standard, namely Conducting and Reporting of Delphi Studies (CREDES) (Junger et al., 2017). The overall process consists of the following three main parts:

### Part 1: Clinical Question Investigation Before the Delphi Consensus Survey

#### Development of the Investigation Questionnaire

In the study, the questions that healthcare professionals mostly care about on making decisions on acupuncture for CI were focused on. The steering group put forward the initial clinical questions on this topic via a brainstorming method and referring to the published literatures or textbooks. Several conferences within the steering group were held for further discussion on these questions. Following multiple revisions, the final version of the questionnaire was sent to the selected acupuncturists for clinical question investigation.

#### Process of Clinical Question Investigation

To provide viable recommendations on the specific issues for acupuncture in the treatment of CI, a clinical panel of 47 Chinese acupuncturists who possessed the professional background and clinical experience were invited. The clinical panel participated into the clinical question investigation as well as gave comments and suggestions simultaneously during the process. Most of the individuals within this panel were clinicians in the field and members of an academic association, namely China Association of Acupuncture-Moxibustion. The detailed demographics of the clinical panel are shown in Table 1. The clinical question investigation was conducted during June 13th to 15th, 2020. A questionnaire consisting of 25 semi-open clinical questions was distributed through a bespoke online software^1^. The predominant contents of the questionnaire were composed of the following four domains: 1) favorable intervention population (3 questions), 2) acupuncture principle and protocol (17 questions), 3) clinical outcomes (3 questions), and 4) adverse events (2 questions). The detailed contents in the questionnaire are shown in Supplementary Table S1. After analyzing and summarizing the results from the returned questionnaires, the items for the Delphi consensus survey were developed according to the PICO (patient, intervention, control, and outcome) framework finally (Robinson et al., 2011), which ensured the inclusion of all critical parts within the clinical question investigation.

**TABLE 1:** Characteristics of the participants.

Variable	Clinical question investigation *n* (% of 47 acupuncturists)	Expert consensus survey *n* (% of 30 experts)
Female	28 (59.57)	11 (36.67)
**Highest education background**		
Bachelor’s degree	4 (8.51)	2 (6.67)
Master’s degree	9 (19.15)	4 (13.33)
Doctor’s degree	34 (72.34)	24 (80)
**Professional title**		
Intermediate title	11 (23.4)	0 (0)
Deputy senior title	18 (38.3)	5 (16.67)
Senior title	18 (38.3)	25 (83.33)
**Acupuncture practical experience (years)**		
3∼10	14 (29.79)	2 (6.67)
11∼20	26 (55.32)	9 (30)
21∼30	7 (14.89)	7 (23.33)
>30	0 (0)	12 (40)
**Institution**		
Hospital or clinic	29 (61.7)	11 (36.67)
Medical school or research institution	18 (38.3)	19 (63.33)
**Geographical distribution**		
East China	11 (23.4)	12 (40)
North China	17 (36.17)	5 (16.67)
South China	2 (4.26)	3 (10)
Central China	8 (17.02)	4 (13.33)
Northeast China	2 (4.26)	3 (10)
Southwest China	5 (10.64)	2 (6.67)
Northwest China	2 (4.26)	1 (3.33)

### Part 2: Collecting the Relevant Evidence in the Field

An electronic literature retrieval within PubMed, EMBASE, and Cochrane Library databases was conducted on June 18th, 2020. The search strategy applied in PubMed is shown in Table 2. Systematic reviews (SRs) on acupuncture in treating CI, restricted to English-language publications, with the full-text obtainable, were included. Literatures were cross-searched by two researchers independently to ensure that all eligible articles could be identified. Using a predesigned information summary table, two assessors extracted information from each paper independently. Grading of Recommendations Assessment, Development and Evaluation (GRADE) tool was applied to assess the methodological quality of evidence in the SRs by two independent researchers. The quality of evidence could be divided into four levels, namely “High,” “Moderate,” “Low,” or “Very low” (Atkins et al., 2004a, b). Inter-assessor discrepancies would be resolved by discussion or arbitration of a third researcher. Subsequently, the extracted research evidence from the included SRs as well as the corresponding GRADE ratings were presented on the expert consensus voting list as reference for experts in making decisions. The experts were allowed to put forward any article they considered was missing.

**TABLE 2:** Search strategy used in PubMed.

No.	Search terms
1	Acupuncture [MeSH Terms] OR acupuncture therapy [MeSH Terms] OR acupuncture points [MeSH Terms] OR acupuncture, ear [MeSH Terms]
2	Acupuncture [Title/Abstract] OR acupuncture therapy [Title/Abstract] OR needle therapy [Title/Abstract] OR acupuncture points [Title/Abstract] OR acupoints [Title/Abstract] OR body acupuncture [Title/Abstract] OR scalp acupuncture [Title/Abstract] OR auricular acupuncture [Title/Abstract] OR ear acupuncture [Title/Abstract] OR manual acupuncture [Title/Abstract] OR electroacupuncture [Title/Abstract] OR electro-acupuncture [Title/Abstract] OR fire needling [Title/Abstract] OR warm needling [Title/Abstract]
3	1 OR 2
4	Dementia [MeSH Terms] OR dementia, multi-infarct [MeSH Terms] OR dementia, vascular [MeSH Terms] OR neurocognitive disorders [MeSH Terms] OR cognition disorders [MeSH Terms] OR cognitive dysfunction [MeSH Terms] OR Alzheimer disease [MeSH Terms]
5	Dementia [Title/Abstract] OR cognitive disorder [Title/Abstract] OR cognitive impairment [Title/Abstract] OR cognitive dysfunction [Title/Abstract] OR cognitive decline [Title/Abstract] OR cognitive degeneration [Title/Abstract] OR Alzheimer disease [Title/Abstract] OR Alzheimer’s disease [Title/Abstract] OR AD [Title/Abstract] OR VD [Title/Abstract] OR VaD [Title/Abstract] OR VCI [Title/Abstract] OR MCI [Title/Abstract] OR SCD [Title/Abstract]
6	4 OR 5
7	Systematic reviews as topic [MeSH Terms] OR meta-analysis as topic [MeSH Terms] OR network meta-analysis [MeSH Terms]
8	Systematic review [Publication Type] OR meta-analysis [Publication Type]
9	Systematic review [Title/Abstract] OR meta-analysis [Title/Abstract] OR network meta-analysis [Title/Abstract]
10	7 OR 8 OR 9
11	3 AND 6 AND 10

### Part 3: Delphi Consensus Survey

#### Sampling of Experts

By searching published literatures and acupuncture textbooks on this topic, the leading authors or editors were identified and taken into consideration as potential candidates. Suitable authoritative experts should own a deputy senior title at least, as well as be fairly familiar with acupuncture in the treatment of neurological diseases. An invitation letter accompanied with the informed consent was sent via e-mail or WeChat (a universal Chinese instant messaging app) beforehand. The study purposes and the details of the survey process were introduced within the letter. After confirmation of their participation by the signed informed consents, the panel of experts took part in the multi-round Delphi consensus survey. Before the formal Delphi survey, several video teleconferences between the expert panel and the steering group were held with the help of Tencent Meeting software to further introduce the whole vote process and answer participants’ queries. In order to ensure confidentiality and independence of judgments during the process, the identities of experts were protected between each other. The experts were required to hold an impartial attitude and make an objective judgment toward each item. Their individual choices were promised to be kept in secret at all times so that they did not need to worry about whether they had contrary opinions with others.

#### Process of the Delphi Consensus Survey

Aiming to facilitate experts for better acquiring the current information of acupuncture in treating CI and making accurate and objective judgment, each item of the survey list would be attached with the results of the clinical question investigation or corresponding content of research evidences from SRs. The experts were instructed to make a selection for each item by integrating the research evidence with their individual knowledge and clinical experience. Comment boxes were also attached so as to provide experts with the opportunity to share their personal advice, if they chose to. These qualitative feedbacks contributed to further modifying the items. In order to ensure the sufficient response rate of the expert panel in each round, experts were required to leave their real names to confirm who had filled in the questionnaire. The consensus survey was conducted during June 28th to August 4th, 2020.

In the first-round survey, an initial voting list including 24 items, as well as the questions about demographic and professional information of experts, was distributed among the participating experts by the same online questionnaire tool (see text footnote 1). A 3-option question including “Agree,” “Neutral,” and “Disagree,” was used to measure the experts’ attitude toward each item (Nava et al., 2019). Panelists were also requested to fill in an explanation for their choice on each item. The results of the Delphi consensus survey were displayed as statistic percentages. Quantitative results were analyzed to decide whether the consensus had been achieved. According to the previous studies of this type, consensus establishment was defined as that the threshold of agreement amongst respondents should be more than 80% (Ravensbergen et al., 2016; Hohmann et al., 2020). As long as a level of agreement was <80%, it suggested that the item was still lack of agreement and required another round of voting to either reach a higher level of agreement or no consensus achievement. In an iterative fashion, the same process would be repeated among the same expert panel. The general voting results, experts’ own choices, and anonymous qualitative comments of the former round would be shown on the new voting list (Wong et al., 2019). This allowed the experts another opportunity to reconsider their choices in view of the whole expert panel’s response. To minimize experts’ fatigue and workload, based on the previous suggestion that a minimum level of agreement should be 70% (Sumsion, 1998), items for which the level of agreement was less than 70% would not be voted again in the next round.

## Results

### Participants in the Expert Panel

A total of 30 experts recruited in China replied and consented to take part in this Delphi consensus survey. The response rates of experts who fulfilled all the 2-round Delphi survey were 100% without dropout during the process. A total of 25/30 experts (83.33%) owned senior title and 28/30 (93.33%) had at least 10 years of acupuncture practical experience. The majority of experts worked in medical schools or research institutions (63.33%), while the others worked in hospitals or clinics. The detailed demographics of the expert consensus panel are shown in Table 1 and Supplementary Table S2. The expert panel authority (Cr) was applied to evaluate the reliability of our study, which depends on two factors, namely the judgment basis (Ca) and familiarity (Cs) of experts. Aiming to acquire the individual values of Ca and Cs, we set two separate questions in the voting list for each expert. The detailed contents and corresponding evaluation criteria are shown in the Supplementary Material. Based on the following formula: Cr = (Ca + Cs)/2, the mean Cr value was 0.86, which suggested that the expert panel authoritative degree was adequate for gaining the desirable opinions (Zhang et al., 2018).

### Evidence in the Field

The initial database search yielded 54 papers in total. Among these, 42 articles were excluded by title and abstract screening. Of the remaining 12 SRs, one Cochrane SR on acupuncture for VCI did not include any clinical trial and was lacking extractable results (Peng et al., 2007). No additional SR was identified by the expert panel members during the voting process. Therefore, 11 eligible SRs were included and analyzed in our study eventually (Lee et al., 2009; Cao et al., 2013; Liu et al., 2014; Zhou et al., 2015, 2017; Deng and Wang, 2016; Kwon et al., 2018; Huang et al., 2019; Kim et al., 2019; Wang et al., 2020; Lai et al., 2020). The contained research evidence of interest was extracted out and further assessed with the help of the GRADE tool. However, the methodological quality of most evidence was rated “Low” or “Very low.” On the other hand, only six items (25%) could be affiliated with the corresponding evidence from these SRs. The other 18 items on the voting list without available evidence were provided with the results from our clinical question investigation as reference. The detailed results extracted from the included SRs are shown in Supplementary Table S3.

### Overall Consensus Status

**FIGURE 2:**
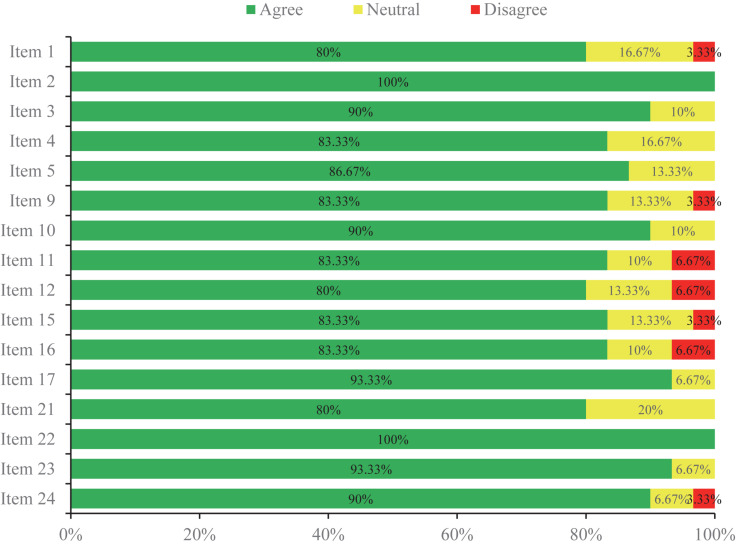
The items which met consensus in Round 1 of the Delphi survey.

**FIGURE 3:**
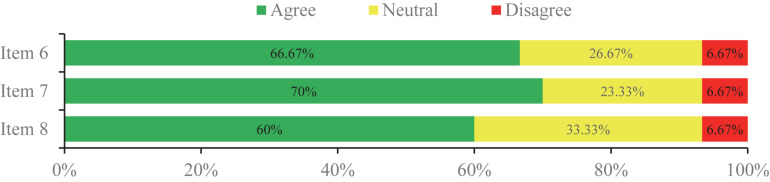
The items that did not reach consensus in the 2-round Delphi survey.

**FIGURE 4:**
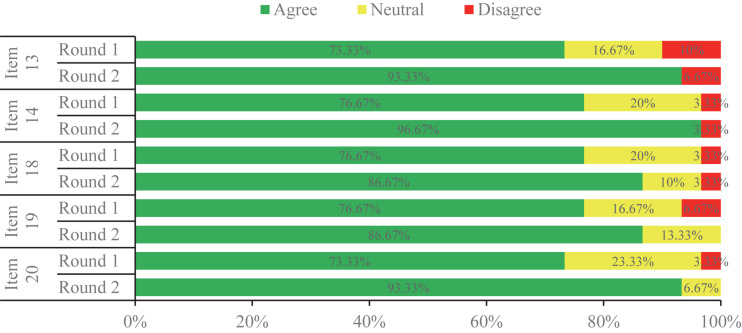
The items which met consensus in Round 2 of the Delphi survey.

In the first round, the expert panel determined the initial 24 items on the voting list generated via the clinical question investigation. There were 16 items (66.67%) which reached a consensus successfully (Figure 2), while three items (12.5%) resulted in poor levels of agreement for consensus (Figure 3). The other five controversial items (20.83%) near consensus in the first round were voted again among the same experts in the second round (Figure 4), which also met the standard for consensus ultimately. Thereinto, in reference to the panelists’ advice, two items (items 19 and 20) were modified without alteration of their original scientific contents before they were moved forward to the second round. According to the experts’ feedbacks on the online questionnaires, no new item was put forward within the 2-round survey. Of the 21 established items, 10 items reached 90∼100% agreement, and 80∼90% expert agreement was achieved for 11 items.

### The Items Voted on in the 2-Round Delphi Consensus Survey

The 24 items to be voted on addressed the relevant and debatable topics on acupuncture in treating CI, of which the 21 items reached a consensus that could be roughly categorized into the six domains: (1) therapeutic effects of acupuncture, (2) therapeutic principles, (3) acupoint selection and combination, (4) acupuncture parameters, (5) considerable combined therapies, and (6) possible adverse events. The detailed statements of all items are shown in Table 3. The specific contents of the consensus reached can be summarized as below:

**TABLE 3:** Summary of the 24 items in the Delphi consensus survey.

No.	Statements	Evidence	GRADE level	Delphi agreement
**(1) Effects of acupuncture for favorable intervention population**				

1	For AD with mild or moderate CI, acupuncture is recommended as an adjuvant therapy to improve cognitive functions (memory, attention, comprehension, linguistic ability, etc.).	Lee et al. (2009), Zhou et al. (2015), Huang et al. (2019), and Wang et al. (2020)	Low/Very low	Round 1
2	For AD with mild or moderate CI, acupuncture is recommended as an adjuvant therapy to improve quality of daily life and ameliorate psychological and mental conditions.	Lee et al. (2009), Zhou et al. (2015), Huang et al. (2019), and Wang et al. (2020)	Low/Very low	Round 1
3	It is recommended to apply acupuncture in the treatment of SCD or MCI.	Cao et al. (2013), Deng and Wang (2016), Kim et al. (2019), and Lai et al. (2020)	Low/Very low	Round 1
4	For mild or moderate VCI, acupuncture is recommended to improve cognitive functions (memory, attention, comprehension, linguistic ability, etc.).	Liu et al. (2014) and Kwon et al. (2018)	Very low	Round 1
5	For mild or moderate VCI, acupuncture is recommended to improve quality of daily life and ameliorate psychological and mental conditions.	Kwon et al. (2018)	Very low	Round 1
6	For PD with mild or moderate CI, acupuncture is recommended as an adjuvant therapy to improve cognitive functions (memory, attention, comprehension, linguistic ability, etc.).			None
7	For PD with mild or moderate CI, acupuncture is recommended as an adjuvant therapy to improve quality of daily life and ameliorate psychological and mental conditions.			None
8	For mild or moderate CI, relief of clinical symptoms can be maintained for 1 to 6 months after one course of acupuncture treatment.			None

**(2) Therapeutic principles**				

9	It is recommended to conduct acupuncture treatment based on syndrome differentiation from syndrome of brain marrow deficiency, syndrome of both *qi* and blood deficiency, syndrome of orifices blocked by phlegm, or syndrome of blood stasis.			Round 1

**(3) Acupoint selection and combination**				

10	It is recommended to select acupoints on Governor Meridian, Kidney Meridian, Heart Meridian, or Bladder Meridian.			Round 1
11	Acupoint selection is a critical factor for the achievement of favorable therapeutic effectiveness of acupuncture. The recommended principal acupoints should be *Baihui* (GV20), *Sishencong* (EX-HN1), and *Shenting* (GV24).			Round 1
12	It is recommended to perform acupoint combination in accordance with syndrome differentiation.			Round 1
13	For CI caused by syndrome of brain marrow deficiency, it is recommended to combine with *Xuanzhong* (GB39) and *Shenshu* (BL23).			Round 2
14	For CI caused by syndrome of both *qi* and blood deficiency, it is recommended to combine with *Zusanli* (ST36) and *Qihai* (RN6).			Round 2
15	For CI caused by syndrome of orifices blocked by phlegm, it is recommended to combine with *Fenglong* (ST40) and *Yinlingquan* (SP9).			Round 1
16	For CI caused by syndrome of blood stasis, it is recommended to combine with *Geshu* (BL17) and *Xuehai* (SP10).			Round 1

**(4) Parameters of acupuncture**				

17	*De-qi* and acupuncture manipulation are the critical factors for the achievement of favorable therapeutic effectiveness of acupuncture.			Round 1
18	It is recommended to select 7∼9 acupoints per session.			Round 2
19	The recommended duration time of needle retention should be 30∼60 min per session.			Round 2
20	Treatment frequency is a critical factor for the achievement of favorable therapeutic effectiveness of acupuncture. The recommended treatment frequency should be 3∼5 times per week.			Round 2
21	Course of treatment is a critical factor for the achievement of favorable therapeutic effectiveness of acupuncture. The recommended course of treatment should be 3 months.			Round 1

**(5) Considerable combined therapies**				

22	It is recommended to combine acupuncture with cognitive training, so as to enhance the clinical effectiveness.			Round 1
23	It is recommended to combine acupuncture with other traditional Chinese medicine therapies, such as Chinese herbal medicine, electroacupuncture, or moxibustion, so as to enhance the clinical effectiveness.	Zhou et al. (2017)	Very low	Round 1

**(6) Possible adverse events**				

24	Adverse event is uncommon in the treatment of CI with acupuncture. The possible adverse events comprise subcutaneous hematoma, local errhysis at acupoints, or abnormal post-acupuncture sensation (such as pain, numbness, etc.).			Round 1

*AD, Alzheimer’s disease; CI, cognitive impairment; MCI, mild cognitive impairment; PD, Parkinson’s disease; SCD, subjective cognitive decline; VCI, vascular cognitive impairment.*

Items 1∼5 refer to the clinical outcomes that acupuncture may produce for the favorable intervention population. The consensus reached by the expert panel in this part is: Acupuncture can be recommended for mild or moderate AD and VCI patients, to improve their cognitive functions and quality of daily life, and ameliorate their psychological and mental conditions. In addition, acupuncture is also recommended to be used in the treatment of SCD or MCI.

Item nine primarily addresses the therapeutic principle of acupuncture, namely the TCM theory based on. The experts recommend that acupuncture therapy should be conducted based on syndrome differentiation from four different kinds of TCM syndromes.

Items 10∼16 comment on the recommended principal acupoints and different acupoint combinations under distinct syndromes. Items 17∼21 analyze the concrete parameters of acupuncture, including requirement for needling sensation, quantity of selected acupoints, time of needle retention, treatment frequency and course, etc. The specific acupuncture procedures recommended for CI in this consensus are: Acupuncturists can take into consideration the selection of acupoints on the four meridians, namely Governor Meridian, Kidney Meridian, Heart Meridian, or Bladder Meridian. *Baihui* (GV20), *Sishencong* (EX-HN1), and *Shenting* (GV24) can be set as the principal acupoints and combined with the other acupoints in accordance with syndrome differentiation. A total of 7∼9 acupoints are preferable to be applied per session. After routine skin disinfection, the needles should be slowly and vertically/horizontally inserted into the corresponding acupoints. The appropriate reinforcing and reducing manipulations of acupuncture technique according to the different syndromes and selected acupoints, involving twirling, lifting, and thrusting can be implemented so as to elicit *De-qi* for patients. Needles can be retained for 30∼60 min per session. The ideal treatment frequency should be 3∼5 times per week and the whole course of treatment should ideally last as long as three months.

Items 22 and 23 discuss the considerable combined therapies with acupuncture for improving the clinical effectiveness. Cognitive training and other TCM therapies are recommended to be used in combination with acupuncture if necessary.

Item 24 considers the general incidence of possible adverse events in the treatment of CI with acupuncture. The expert panel members believe that adverse events are uncommon in the treatment of CI with acupuncture.

## Discussion

In this study, a panel of veteran acupuncture experts were invited to provide some viable recommendations on the specific issues for acupuncture practitioners in treating CI. Eventually, after a 2-round Delphi survey, the expert consensus of acupuncture therapy for CI amongst the participants achieved high levels of agreement for the majority of items (21/24). Consensus was established in six domains under investigation. Given the academic background of acupuncture, several items are specific to the constructs of Traditional Chinese Meridian theory. Nonetheless, this expert consensus can still provide some practical and generalized recommendations for clinical acupuncturists.

According to the final achieved consensus items, more than 80% of the experts agree that acupuncture can be used to improve cognitive functions of patients with mild or moderate CI caused by AD or cerebrovascular diseases, which has been underpinned by the results of previous studies (Liu et al., 2014; Wang et al., 2020). However, the paucity of clinical trials exploring the efficacy of acupuncture for dementia owing to Parkinson’s disease (PD) precluded consensus on this topic. Even though some experts believed that PD is another common disease suitable for acupuncture application in their clinical practice and acupuncture contributes to motor symptom improvement (Kabra et al., 2018), whether CI due to PD could be changed by acupuncture has not been paid close attention to. In view of the lack of corresponding evidence and clinical experience at present, they had to maintain a neutral or opposite attitude. Meanwhile, most of the experts considered that it was an interesting issue and researchers could strive to design and carry out new rigorous prospective randomized clinical trials (RCTs) aiming to prove acupuncture to be therapeutically valuable in this kind of dementia. In addition, SCD and MCI are relatively milder than dementia and always regarded as the prodromal stages of AD. Without timely and effective intervention, these patients have a higher tendency to progress to meet the diagnostic criteria of dementia than their healthy peers (Petersen et al., 2001; Stewart, 2012). Given that there still exists plasticity at these early stages, the expert panel reached almost unanimous agreement that acupuncture should be encouraged as a potential treatment in SCD and MCI for preventing further progression. On the other hand, accompanied with the deterioration of cognitive function in CI patients, their abilities to perform activities of daily life and psychological conditions are also significantly affected (Desai et al., 2004). Acupuncture’s effect in improving CI patients’ quality of life has been recognized by our experts and shown in the published SRs (Zhou et al., 2015; Kwon et al., 2018). Although the experts also agree that acupuncture is able to relieve psychiatric disorders, given the lack of corresponding evidence, we suggest that outcomes of mental state can be focused on more in future studies. As for how long these therapeutic effects of acupuncture can last, the expert panel reached no consensus eventually. A major reason is the absence of available evidence for them to make an exact judgement. Moreover, several experts frankly confessed that they did not conduct follow-up seriously beforehand and were inexperienced. This controversial issue highlights the gap that urgently needs to be filled by new clinical trials and the long-term effectiveness of acupuncture which should be focused on.

Based on the classical Meridian theory, acupoints are the specially defined points on the body surface and play a crucial role in determining acupuncture effect (Xing et al., 2013). Both acupoint selection and combination are the core parts of acupuncture treatment. The recommended principal acupoints for CI in this consensus are *Baihui* (GV20), *Sishencong* (EX-HN1), and *Shenting* (GV24), which is concordant with the founding of SRs that these acupoints are the frequently adopted acupoints in previous studies (Zhou et al., 2015; Wang et al., 2020). Although the three principal acupoints are always taken into consideration in the treatment of neurological diseases and supported by the TCM theory, further clinical trials are still needed to confirm their superiority over other acupoints in treating CI. Tailoring diagnosis and treatment to the individual is one of the basic principles and unique virtues of acupuncture (Cao et al., 2012). The method of acupoint combination in accordance with syndrome differentiation has reached a high level of agreement amongst the experts. By syndrome differentiation, CI can be further subdivided into the four different subtypes. After mastering the accurate syndrome subtype, the acupuncturist can choose one from the four distinct kinds of acupoint combinations, which were also approved by the majority of the experts. In spite of the fact that treatment based on syndrome differentiation is emphasized by acupuncture practitioners, most RCTs applied completely uniform protocols for CI patients but did not take their syndrome subtypes into account. To the best of our knowledge, there is no research exploring whether this concept does contribute to achieving more favorable effectiveness in acupuncture treatment for CI. Thus, we suggest that not only clinical practice, but also new study designing may refer to these expert consensus items.

Different from other pharmacological therapies, acupuncture is a kind of sophisticated intervention whose therapeutic effects can be influenced by a series of factors (Shi et al., 2012). It has been suggested by published literatures that the clinical outcomes produced by acupuncture may be dose-dependent and hinged on an appropriate acupuncture protocol (White et al., 2007). In addition, the clinical acupuncturists also have their own accumulated experience for gaining better efficacy by means of daily practice, which may be worthwhile to summarize and analyze (Lin et al., 2016). All of these variabilities among existing different clinical trials might cast uncertainty on the actual effectiveness of acupuncture for CI. Nevertheless, this issue is always neglected by most RCTs that often focus much more on validity assessment but are less concerned with the ideal protocol (Sun et al., 2020). Therefore, there were five items on the relevant acupuncture parameters which were voted on to explore the proper acupuncture stimulation for ensuring the ideal therapeutic effects. Of note, many experts laid stress on the significance of adequate stimulus intensity of acupuncture. They strongly believed that longer needle retention time (especially for scalp acupuncture), more frequent acupuncture (three sessions per week at least), and intact treatment course (lasting for 3 months) contribute to the enhancement of favorable effectiveness of acupuncture for CI. Even though these recommended acupuncture parameters in the consensus statement have been recognized by most of the expert panel members, we still suggest more studies to verify their reliability in the future. Moreover, in order to improve general effectiveness for CI, cognitive training and other TCM therapies, such as Chinese herbal medicine, electroacupuncture, or moxibustion are preferable for combining with manual acupuncture, which conforms to the concept that synthetic remedy can be more beneficial for CI rehabilitation (Loewenstein et al., 2004; Zhou et al., 2017).

In most cases, CI is caused by chronic and progressive diseases, which means that CI patients always ask for a continuous treatment. On this account, there is no wonder that the safety issue should be of great concern among patients and clinicians. Patients have to be confronted with the potential risk of drug-induced side effects, particularly after long-term utilization (Kavirajan and Schneider, 2007). The expert panel agrees that adverse event is uncommon and generally acceptable in the treatment of CI with acupuncture, which is another superiority of acupuncture demonstrated by previous SRs (Kim et al., 2019; Wang et al., 2020). Therefore, acupuncture, as a relatively safe treatment modality, may be worthy of consideration for patients suffering from CI for a long time.

The value of this kind of study for clinical practice is arguable. As an indispensable part of evidence-based medicine, RCTs are commonly regarded as the gold standard to measure the outcomes produced by a certain intervention, but also accompanied with some immanent drawbacks (Hohmann et al., 2018a). Subject to the rigorous inclusion and exclusion criteria of the study population, high methodologic requirements, as well as resource intensive with respect to time and cost, they may not fully meet the needs for general clinical practice (Atkins et al., 2004a; Frieden, 2017; Hohmann et al., 2018a). For instance, several potential focuses of actual interest, such as the optimal parameters of acupuncture and acupoint selection closely correlating with clinical treatment, have not been resolved by the previous papers. These existent complicated clinical issues are hardly solvable by RCT studies and SRs in a short time, but the answers to the actual questions in clinic are what acupuncturists urgently concern about. Different from RCTs or SRs, following a structural process to collect controlled response for achieving agreement on specific issues, expert consensus study can synthesize authoritative expert viewpoints in a scientific pattern of high quality (Hohmann et al., 2018a). In addition, the Delphi technique has a specific superiority that it can even be carried out securely while current research evidence is insufficient to determine the answer of an uncertain field. The current study, indeed, provides another optional method to explore more direct solutions for certain important but controversial and intricate clinical questions (Powell, 2003; Keeney et al., 2006; Sandrey and Bulger, 2008). Furthermore, because the success of acupuncture therapy depends on a series of essential characteristics, the expert opinions from this consensus may also be helpful in designing the ideal acupuncture protocols for new high-quality and large-scale RCTs so as to achieve positive results. Nevertheless, aiming to perform this survey more comprehensively and follow the rigorous methodology, prior to the formal expert consensus survey, we carried out the clinical question investigation to systematically collect the topics of interest among the frontline acupuncturists from diverse areas of China. Simultaneously, we still sought evidence from SRs and presented it as well as the results from clinical question investigation to provide the expert panel with more information in making decisions.

It might be argued that the strategy of expert sampling and the issue that evidence is scarce (or absent) for many items under discussion in our study. We agree that a multidisciplinary expert board would be necessary for generating an objective consensus. Consensus reached among the expert board including the individuals who are professionals in the field of CI but do not necessarily practice acupuncture can achieve the higher credibility. However, this survey is about a complicated intervention whose major purpose is to seek some pragmatic answers from the authoritative experts for the specific clinical issues under the circumstance that the relevant evidence is really too scarce. More than 60% of the items voted in the consensus survey are closely related to the concrete acupuncture protocol that are necessary to be standardized and optimized. These recommendations are also what acupuncture practitioners want to learn about in daily practice. Without the referable evidence, these items have to be discussed among experts with background and actual clinical experience on acupuncture. It is difficult for the experts who have never practice acupuncture in CI’s treatment to make a judgment for these actual issues, especially in the absence of or lack of available evidence from the published literatures. Hence, only reputable acupuncture experts were recruited in our study. Nonetheless, in order to reduce the potential bias as much as possible, we also set a more rigorous criterion (80% agreement) for the consensus achievement in our study, which is usually 70% in many other expert consensus surveys. On the other hand, evidence is indeed very essential in establishing guidelines for the clinical practitioners. For a long time, the alternative/complementary techniques are criticized and more subjected to base on ideology, beliefs, and personal experience, rather than on proper and well-built evidence. Even though a lot of acupuncture clinical trials have emerged in the recent decades, most of these studies only focus on assessing the efficacy of acupuncture, and the fact that acupuncture is one kind of sophisticated intervention whose therapeutic effects can be influenced by a series of factors is always overlooked. The concrete acupuncture protocols vary enormously in different literatures which may confound the interpretation of their results. Due to scarce or absent evidence in the fields, the comprehensive experts’ opinions collected with the assistance of the Delphi method may provide another source of reference for acupuncturists to base their treatment on at present. Although many items in this consensus are the specialist’s recommendations rather than a guideline, and their validities still need further verification, these items can point out the reference directions for the future studies at least, and help researchers know what needs to be taken into consideration in better designing the new study protocol and which optimal acupuncture parameters should be further explored.

There are several acknowledged limitations in this study. First, only Chinese rather than global experts took part in the survey, even though they ranged over most regions of China. Therefore, the transferability may be impeded by the confined background of participants. In addition, despite elaborative expert panel sampling and the rigorous Delphi technique followed aiming at the best possible synthesis, the consensus still cannot represent the overall acupuncture community’s opinion. Second, the majority of the items achieved consensus on grounds of the subjective opinions and individual clinical experience of experts. We cannot rule out that panelists might have their own interpretation toward the items, either. The expert opinion is criticized as the lowest valuable denominator of evidence level system (Level V) (Phillips, 2004). Hence, agreement of a certain item is at a particular timepoint and may be altered with emerging new evidence and experience. Third, in spite of the existing differences between doctors’ and patients’ opinions toward one therapy (Luedtke et al., 2020), our clinical question investigation was conducted merely among clinicians, which means the consensus was ground on doctors’ general perspective but not patient specific.

Given the limitations mentioned above, some items in the current survey are more an incipient dialectical consensus than a proper evidence-based guideline to some degree, which need to be viewed with extreme caution by the readers. Notwithstanding, we still look forward to more proper RCTs and robust SRs which can verify the experts’ recommendations and further underlay an ideal expert consensus or clinical guidelines. When more emerging evidence can be provided in the future, an updated version of a multidisciplinary, international, and thoroughly evidence-based expert consensus survey will be feasible and indispensable.

## Conclusion

It is urgent to provide plenty of CI patients with safer and more cost-effective therapeutic options, and acupuncture is one of the potential interventions. Therefore, we conducted this expert consensus survey to provide a certain reference for future clinical practice under the circumstance that current research evidence is relatively insufficient to establish a clinical guideline. The proposed items are not claimed to be the best or most correct ones, but do provide acupuncturists with some pragmatic recommendations on which to base their treatment.

## Data Availability Statement

The original contributions presented in the study are included in the article/Supplementary Material, further inquiries can be directed to the corresponding author.

## Ethics Statement

Ethical review and approval was not required for the study on human participants in accordance with the local legislation and institutional requirements. The participants provided their written informed consent to participate in this study.

## Author Contributions

X-TS and C-ZL put forward the idea of performing this study. X-TS wrote the initial manuscript. L-QW and LW conducted the literature retrieval and evidence extraction. J-LL drew the figures. NZ summarized the tables. G-XS and J-WY further modified and polished the article. All authors participated into the discussion and approved this submitted manuscript.

## Conflict of Interest

The authors declare that the research was conducted in the absence of any commercial or financial relationships that could be construed as a potential conflict of interest.

## Footnotes

1 www.wjx.cn
