# Supplementary material for: Acupuncture Therapy for Cognitive Impairment: A Delphi Expert Consensus Survey
Source: Front Aging Neurosci. 2020 Nov 26;12:596081. doi: 10.3389/fnagi.2020.596081 (PMC7732673; doi:10.3389/fnagi.2020.596081)
Supplement: Supplementary file 1 [file Table_1.pdf]

Supplementary Material

Acupuncture Therapy for Cognitive Impairment: A Delphi Expert Consensus Survey

Xin-Tong Su[1], Li-Qiong Wang[1], Jin-Ling Li[1], Na Zhang[2], Lu Wang[1], Guang-Xia Shi[1], Jing-Wen Yang[1] and Cun-Zhi Liu[1*]

[1] *International Acupuncture and Moxibustion Innovation Institute, School of Acupuncture-Moxibustion and Tuina, Beijing University of Chinese Medicine, Beijing, China*

[2] *School of Acupuncture, Moxibustion and Tuina, Shandong University of Traditional Chinese Medicine, Jinan, China*

Table S1. The 25 questions in the questionnaire of clinical question investigation

NO.	Questions
**Favorable intervention population**	
1	According to the pathological causes, what kinds of CI may acupuncture be applied for?
2	According to the phases of disease progression, what stages of CI may acupuncture be applied in?
3	In reference to the MMSE scale, what severities of CI may acupuncture be applied for?
**Acupuncture principle and protocol**	
4	Do you think it is essential to conduct acupuncture treatment for CI based on syndrome differentiation?
5	If you think it is essential to conduct acupuncture treatment for CI based on syndrome differentiation, what kinds of TCM syndromes can acupuncture be applied for?
6	Do you think it is essential to conduct acupuncture treatment for CI based on meridian diagnosis?
7	If you think it is essential to conduct acupuncture treatment for CI based on meridian diagnosis, which meridians can be used to choose acupoints from?
8	Which 2~3 principal acupoints do you mostly use in the treatment of CI?

9	Which method of acupoint combination should be used in the treatment of CI?
10	If you think it is appropriate to perform acupoint combination in accordance with syndrome differentiation, which acupoints can be combined with for CI caused by syndrome of brain marrow deficiency?
11	If you think it is appropriate to perform acupoint combination in accordance with syndrome differentiation, which acupoints can be combined with for CI caused by syndrome of both *qi* and blood deficiency?
12	If you think it is appropriate to perform acupoint combination in accordance with syndrome differentiation, which acupoints can be combined with for CI caused by syndrome of orifices blocked by phlegm?
13	If you think it is appropriate to perform acupoint combination in accordance with syndrome differentiation, which acupoints can be combined with for CI caused by syndrome of blood stasis?
14	Which kinds of factors are the critical ones for the achievement of favorable therapeutic effectiveness of acupuncture in CI?
15	How many acupoints per session do you mostly use in the treatment of CI?
16	How long should the needles be retained for in the treatment of CI?
17	Which treatment frequency do you mostly set in the treatment of CI?
18	How long the course of treatment should be set in the treatment of CI?
19	In order to enhance the clinical effectiveness, do you think it is necessary to combine acupuncture with other intervention? Which kinds of intervention would you recommend?
20	If you think it is helpful in improving the clinical effectiveness to combine acupuncture with other TCM therapies, which kinds of TCM therapies would you recommend?
**Clinical outcomes**	
21	Which kinds of clinical outcomes may be ameliorated by acupuncture in the treatment of CI?
22	Which kinds of cognitive functions may be improved by acupuncture in the treatment of CI?
23	How long the effectiveness of acupuncture may be maintained for after one course of treatment for CI?
**Adverse events**	
24	What are the possible adverse events in the treatment of CI with acupuncture?
25	Do you think adverse event is uncommon in the treatment of CI with acupuncture?

Abbreviations: CI: cognitive impairment; MMSE: mini mental state examination; TCM: Traditional Chinese Medicine.

Table S2. Expert consensus panel members

NO.	Name	Institution
1	Bin Xu	Nanjing University of Chinese Medicine
2	Neng-Gui Xu	Guangzhou University of Chinese Medicine
3	Shu Wang	The First Teaching Hospital of Tianjin University of Traditional Chinese Medicine
4	Shu-Guang Yu	Chengdu University of Chinese Medicine
5	Yan-Jun Du	Hubei University of Chinese Medicine
6	Sheng-Feng Lu	Nanjing University of Chinese Medicine
7	Tie-Ming Ma	Liaoning University of Traditional Chinese Medicine
8	Bo Chen	Tianjin University of Traditional Chinese Medicine
9	Mei Lu	Henan University of Chinese Medicine
10	Feng-Xia Liang	Hubei University of Chinese Medicine
11	Jun Yang	The First Affiliated Hospital of Anhui University of Chinese Medicine
12	Xing-Ke Yan	Gansu University of Chinese Medicine
13	Min Li	Guangzhou University of Chinese Medicine
14	Hua Sun	Peking Union Medical College Hospital
15	Yong-Jun Peng	Jiangsu Province Hospital of Chinese Medicine
16	Jin-Xia Ni	Dongzhimen Hospital of Beijing University of Chinese Medicine
17	Xin-Jun Wang	Nanjing University of Chinese Medicine
18	Xue-Yong Shen	Shanghai University of Traditional Chinese Medicine
19	Wen-Bin Fu	Guangdong Province Hospital of Traditional Chinese Medicine
20	Shuang Zhou	Shanghai University of Traditional Chinese Medicine
21	Hong Zhang	Chengdu University of Chinese Medicine
22	You-Bing Xia	Xuzhou Medical University
23	Xian-Ming Lin	The Third Affiliated Hospital of Zhejiang Chinese Medical University

24	Jun Xiong	The Affiliated Hospital of Jiangxi University of Traditional Chinese Medicine
25	Yuan-Hao Du	The First Teaching Hospital of Tianjin University of Traditional Chinese Medicine
26	Tian-Song Yang	The First Affiliated Hospital of Heilongjiang University of Chinese Medicine
27	Zhong-Yu Zhou	Hubei Provincial Hospital of Traditional Chinese Medicine
28	Yong-Qing Yang	Shanghai University of Traditional Chinese Medicine
29	Mei-Qi Zhou	Anhui University of Chinese Medicine
30	Hong-Na Yin	The Second Affiliated Hospital of Heilongjiang University of Chinese Medicine

Table S3. Results extracted from the included systematic reviews

NO.	Results extracted from the included systematic reviews
1	**For AD,**<br>(i) MMSE:<br>Acupuncture plus drug therapy vs. drug therapy: 14 RCTs; MD 2.96; 95% CI 1.8, 4.13; $p < 0.01$ [GRADE: Very low] (Wang et al., 2020).<br>Electroacupuncture vs. drug therapy: 2 RCTs; MD -0.55; 95% CI -1.31, 0.21; $p = 0.15$; [GRADE: Low] (Lee et al., 2009).<br>Acupuncture vs. drugs: 6 RCTs; MD 1.05; 95% CI 0.16, 1.93; $p = 0.02$; [GRADE: Very low] (Zhou et al., 2015).<br>Acupuncture plus donepezil vs. donepezil: 3 RCTs; MD 2.37; 95% CI 1.53, 3.21; $p < 0.00001$; [GRADE: Very low] (Zhou et al., 2015).<br>Acupuncture vs. western drugs: 10 RCTs; MD 1.96; 95% CI 0.66, 3.26; $p = 0.003$; [GRADE: Very low] (Huang et al., 2019).<br>(ii) ADAS-cog:<br>Acupuncture vs. drug therapy: 4 RCTs; MD -2.56; 95% CI -4.57, -0.55; $p = 0.01$; [GRADE: Very low] (Wang et al., 2020).

	Acupuncture plus drug therapy vs. drug therapy: 5 RCTs; MD -3.21; 95% CI -5.53, -0.89; p = 0.02; [GRADE: Very low] (Wang et al., 2020).<br>(iii) Response rate:<br>Acupuncture vs. western drugs: 12 RCTs; RR 1.17; 95% CI 1.06, 1.29; p = 0.001; [GRADE: Very low] (Huang et al., 2019).
2	**For AD,**<br>(i) ADL:<br>Acupuncture vs. drug therapy: 11 RCTs; MD 0.21; 95% CI -0.74, 1.16; p = 0.66; [GRADE: Very low] (Wang et al., 2020).<br>Acupuncture plus drug therapy vs. drug therapy: 7 RCTs; MD -1.87; 95% CI -3.17, -0.57; p < 0.01; [GRADE: Very low] (Wang et al., 2020).<br>Electroacupuncture vs. drug therapy: 2 RCTs; MD -1.29; 95% CI -1.77, -0.8; p < 0.001; [GRADE: Low] (Lee et al., 2009).<br>Acupuncture vs. drugs: 4 RCTs; MD -2.80; 95% CI -4.57, -1.02; p = 0.002; [GRADE: Very low] (Zhou et al., 2015).<br>Acupuncture plus donepezil vs. donepezil: 2 RCTs; MD -2.64; 95% CI -4.95, -0.32; p = 0.03; [GRADE: Very low] (Zhou et al., 2015).<br>Acupuncture vs. western drugs: 8 RCTs; MD -1.99; 95% CI -3.34, 0.65; p = 0.004; [GRADE: Very low] (Huang et al., 2019).
3	**For amnestic MCI, MCI in AD, or vascular MCI,**<br>(i) MMSE:<br>Acupuncture vs. nimodipine: 3 RCTs; MD 0.99; 95% CI 0.71, 1.28; p < 0.01; [GRADE: Very low] (Deng and Wang, 2016).<br>Acupuncture plus nimodipine vs. nimodipine: 2 RCTs; MD 0.88; 95% CI 0.28, 1.49; p < 0.01; [GRADE: Very low] (Deng and Wang, 2016).<br>Acupuncture plus other treatment vs. other treatment: 6 RCTs; MD 1.99; 95% CI 1.09, 2.88; p < 0.0001; [GRADE: Very low] (Cao et al., 2013).<br>Electroacupuncture vs. western medications: 5 RCTs; MD 0.65; 95% CI 0.28, 1.01; p = 0.0005; [GRADE: Very low] (Kim et al., 2019).<br>Acupuncture vs. placebo:<br>Direct MA: 2 RCTs; MD 1.04; 95% CI 0.56, 1.52; [GRADE: Low] (Lai et al., 2020).

	Network MA: 23 RCTs; SMD 1.22; 95% CI -0.97, 3.39; [GRADE: Low] (Lai et al., 2020).<br>(ii) MoCA:<br>Electroacupuncture vs. western medications: 2 RCTs; MD 0.66; 95% CI 0, 1.32; $p = 0.05$; [GRADE: Very low] (Kim et al., 2019).<br>(iii) ADAS-cog:<br>Direct MA: 2 RCTs; MD -1.36; 95% CI -1.44, -1.28; [GRADE: Low] (Lai et al., 2020).<br>Network MA: 17 RCTs; SMD -2.84; 95% CI -5.61, -0.34; [GRADE: Low] (Lai et al., 2020).<br>(iv) Response rate:<br>Acupuncture vs. nimodipine: 3 RCTs; OR 1.78; 95% CI 1.19, 2.65; $p < 0.01$; [GRADE: Very low] (Deng and Wang, 2016).<br>Acupuncture plus nimodipine vs. nimodipine: 1 RCT; OR 4.57; 95% CI 1.92, 10.9; $p < 0.01$; [GRADE: Very low] (Deng and Wang, 2016).
4	**For VCI,**<br>(i) MMSE:<br>Auricular acupuncture vs. western medications: 3 RCTs; MD 0.73; 95% CI −0.02, 1.48; $p = 0.06$; [GRADE: Very low] (Kwon et al., 2018).<br>Acupuncture vs. medicine or rehabilitation: 4 RCTs; MD 3.14; 95% CI 2.06, 4.21; $p < 0.00001$; [GRADE: Very low] (Liu et al., 2014).
5	**For VCI,**<br>(i) ADL:<br>Auricular acupuncture vs. western medications: 2 RCTs; MD 0.2; 95% CI −3.51, 3.91; $p = 0.92$; [GRADE: Very low] (Kwon et al., 2018).
23	**For AD, acupuncture plus Chinese herbal medicine vs. western drugs,**<br>(i) MMSE:<br>11 RCTs; MD 2.1; 95% CI 0.69, 3.51; $p = 0.004$; [GRADE: Very low] (Zhou et al., 2017).<br>(ii) ADL:<br>3 RCTs; MD -3.59; 95% CI -7.18, 0.01; $p = 0.05$; [GRADE: Very low] (Zhou et al., 2017).<br>(iii) Response rate:<br>12 RCTs; OR 2.72; 95% CI 2.04, 3.62; $p < 0.00001$; [GRADE: Very low] (Zhou et al., 2017).<br>(iv) Traditional Chinese Medicine symptom score:<br>3 RCTs; MD 5.07; 95% CI 3.9, 6.25; $p < 0.0001$; [GRADE: Very low] (Zhou et al., 2017).

Abbreviations: AD: Alzheimer's disease; ADAS: Alzheimer's disease assessment scale; ADL: activity of daily life; CI: confidence interval; GRADE: Grading of Recommendations Assessment, Development and Evaluation; MA: meta-analysis; MCI: mild cognitive impairment; MD: mean difference; MMSE: mini mental state examination; MoCA: Montreal cognitive assessment; OR: odds ratio; RCTs: randomized clinical trials; RR: risk ratio; SCD: subjective cognitive decline; SMD: standardized mean difference; VCI: vascular cognitive impairment.

The two questions and corresponding evaluation criteria to determine the values of judgement basis (Ca), familiarity (Cs), and expert authority (Cr)

We calculated the mean authority (Cr) value after gathering the Cr values of the individual experts. For each expert, Cr = (Ca + Cs)/2.

(i) Are you familiar with acupuncture therapy for cognitive impairment?

Familiarity	Very familiar	Familiar	General	Unfamiliar	Very unfamiliar
Cs value	1.0	0.8	0.5	0.2	0

(ii) What is your major judgement basis for the items on the voting list?

Judgement basis	Literatures	Clinical experience	Theoretical analysis	Peer opinions	Individual intuition
Ca value	0.9	0.8	0.6	0.4	0.2

References

Cao, H., Wang, Y., Chang, D., Zhou, L., and Liu, J. (2013). Acupuncture for vascular mild cognitive impairment: A systematic review of randomised controlled trials. *Acupunct Med*. 31, 368-374. doi: 10.1136/acupmed-2013-010363

Deng, M., and Wang, X.F. (2016). Acupuncture for amnestic mild cognitive impairment: A meta-analysis of randomised controlled trials. *Acupunct Med*. 34, 342-348. doi: 10.1136/acupmed-2015-010989

Huang, Q., Luo, D., Chen, L., Liang, F.X., and Chen, R. (2019). Effectiveness of acupuncture for Alzheimer's disease: An updated systematic review and meta-analysis. *Curr Med Sci*. 39, 500-511. doi: 10.1007/s11596-019-2065-8

Kim, H., Kim, H.K., Kim, S.Y., Kim, Y.I., Yoo, H.R., and Jung, I.C. (2019). Cognitive improvement effects of electro-acupuncture for the treatment of MCI compared with Western medications: A systematic review and Meta-analysis. *BMC Complement Altern Med*. 19, 13. doi: 10.1186/s12906-018-2407-2

Kwon, C.Y., Lee, B., Suh, H.W., Chung, S.Y., and Kim, J.W. (2018). Efficacy and safety of auricular acupuncture for cognitive impairment and dementia: A systematic review. *Evid Based Complement Alternat Med*. 2018, 3426078. doi: 10.1155/2018/3426078

Lai, X., Wen, H., Li, Y., Lu, L., and Tang, C. (2020). The comparative efficacy of multiple interventions for mild cognitive impairment in Alzheimer's disease: A Bayesian network Meta-Analysis. *Front Aging Neurosci*. 12, 121. doi: 10.3389/fnagi.2020.00121

Lee, M.S., Shin, B.C., and Ernst, E. (2009). Acupuncture for Alzheimer's disease: A systematic review. *Int J Clin Pract*. 63, 874-879. doi: 10.1111/j.1742-1241.2009.02043.x

Liu, F., Li, Z.M., Jiang, Y.J., and Chen, L.D. (2014). A meta-analysis of acupuncture use in the treatment of cognitive impairment after stroke. *J Altern Complement Med*. 20, 535-544. doi: 10.1089/acm.2013.0364

Wang, Y.Y., Yu, S.F., Xue, H.Y., Li, Y., Zhao, C., and Jin, Y.H. (2020). Effectiveness and safety of acupuncture for the treatment of Alzheimer's disease: A systematic review and Meta-Analysis. *Front Aging Neurosci*. 12, 98. doi: 10.3389/fnagi.2020.00098

Zhou, J., Peng, W., Xu, M., Li, W., and Liu, Z. (2015). The effectiveness and safety of acupuncture for patients with Alzheimer disease: A systematic review and meta-analysis of randomized controlled trials. *Medicine (Baltimore)*. 94, e933. doi: 10.1097/MD.0000000000000933

Zhou, S., Dong, L., He, Y., and Xiao, H. (2017). Acupuncture plus herbal medicine for Alzheimer's disease: A systematic review and Meta-Analysis. *Am J Chin Med*. 45, 1327-1344. doi: 10.1142/S0192415X17500732
